# Gallstones in Patients with Chronic Liver Diseases

**DOI:** 10.1155/2017/9749802

**Published:** 2017-01-31

**Authors:** Xu Li, Xiaolin Guo, Huifan Ji, Ge Yu, Pujun Gao

**Affiliations:** Department of Hepatology, The First Hospital of Jilin University, Jilin University, Changchun, China

## Abstract

With prevalence of 10–20% in adults in developed countries, gallstone disease (GSD) is one of the most prevalent and costly gastrointestinal tract disorders in the world. In addition to gallstone disease, chronic liver disease (CLD) is also an important global public health problem. The reported frequency of gallstone in chronic liver disease tends to be higher. The prevalence of gallstone disease might be related to age, gender, etiology, and severity of liver disease in patients with chronic liver disease. In this review, the aim was to identify the epidemiology, mechanisms, and treatment strategies of gallstone disease in chronic liver disease patients.

## 1. Introduction 

Gallstones (GS), which were first described in 1507 by Antonio Benivenius [1], are abnormal masses of a solid mixture of cholesterol crystals, calcium carbonate, phosphate, bilirubinate, and palmitate, phospholipids, glycoproteins, and mucopolysaccharides that have affected people for centuries. With prevalence of 10–20% in adults in developed countries [2–4], GS disease (GSD) is one of the most prevalent and costly gastrointestinal tract disorders in the world. The growing global epidemic of obesity and metabolic syndrome will likely increase the rates of GSD worldwide [5].

In addition to GSD, chronic liver disease (CLD) is also an important global public health problem. The reported frequency of GSD in CLD tends to be higher [6–8]. Sheen and Liaw [9] suggested that CLD is a risk factor for cholecystolithiasis through their study of the prevalence and incidence of cholecystolithiasis in CLD patients. Because the clinical manifestations of gallstones are similar to those of CLD and it is problematic to apply laparoscopic cholecystectomy to assess liver malfunction, the management of GSs in patients with CLD remains difficult [2–4, 10], despite the high incidence of both CLD and GSD. In this review, the aim was to identify the epidemiology, mechanisms, and treatment strategies of GSD in CLD patients.

## 2. Epidemiology 

GSs are found in 10–20% of the general adult population [2–4]. The prevalence of the clinical manifestation of GS in patients with CLD is largely unknown and was initially assessed in autopsy studies [11–15]. In general, the frequency of GS in patients with CLD ranges from 3.6% to 46%, with a 1.2- to 5-fold increase compared with the general population [2–4, 9, 12, 16] (Table 1). Most case-control studies based on ultrasound findings found that prevalence of cholecystolithiasis was significantly higher in patients with liver cirrhosis than in controls [9, 17]. A recent prospective ultrasound study of medical records of 500 patients with various forms of liver cirrhosis found the prevalence of GS to be 29.4% [16]. In a prospective study of 618 patients with liver cirrhosis followed for almost 4 years, during this period, the incidence of new GS which was monitored ultrasonographically was 22.8% [18]. Similarly, Acalovschi et al. [7] found that even CLD patients but not those with cirrhosis had higher prevalence of GS (diagnosed by ultrasonography) than healthy people.

There exists 3 : 1 female predominance for the prevalence of GS in patients without CLD [3, 4, 19], but the prevalence of GS in the population with CLD is still unclear [20–23]. In some reports, the female-to-male ratio in CLD patients was similar in GS carriers without CLD [9, 20, 24], while some other studies showed that the female-to-male ratio approached 1 : 1 in CLD patients [12, 16, 17, 19, 25–27]. Cirrhosis was shown to represent a risk factor for GS in men but not in women [17, 23], and male gender was identified as an independent risk factor for GSD [28]. The reason for the sex-specific differences between CLD and GSD are likely due to the increased levels of progesterone and estrogen in males with CLD, which can impair gallbladder emptying as observed in pregnant women [17].

Conte et al. [18] found that the prevalence of GS increased significantly with age. Some other studies also showed that GS prevalence increases with age in CLD patients [6, 14, 20, 29, 30]. But others did not observe the linear trend of increasing prevalence with increasing age in CLD patients [9, 20, 27]. In one of these studies, Coelho et al. studied a series of 400 cirrhotic patients undergoing liver transplantation in Brazil; they showed that the prevalence in GSD increased with age in the transplant recipients [27]. Considering the mechanisms of GS formation in cases of CLD [31–34], such as oversecretion of bilirubin due to increased hemolysis secondary to hypersplenism [35, 36] and impaired reduction of phospholipid and bile acid secretion compared to cholesterol secretion [36], it seems possible that GSD could develop in a young CLD patient and that the influence of liver disease on the prevalence of GSD is stronger than that of age.

## 3. Incidence according to Etiology of Liver Disease and Stage of Liver Disease

### 3.1. Viral Infection

The association between hepatic viral infection and GSD has been evaluated in several studies. There are several possible mechanisms for the relationship between hepatitis virus and GS formation, such as the direct infection of the gallbladder by the virus. Sulaberidze et al. [37] showed that the cholecystopathogenic influence of the HBV leads to structural and functional changes of the gallbladder.

Increased prevalence of GSD was associated with the duration and severity of HBV-related liver disease [9]. This means that the risk of cholelithiasis increases over time in patients with HBV. In addition, in a study performed in the United States, Bini and McGready found that chronic HCV infection was strongly associated with gallbladder disease among men [38].

Lee et al. [39] showed that HBV and HCV were associated with GSD in the elderly. The direct infection of the gallbladder by HCV may also play an important role in the development of GS. Loriot et al. [40] found the HCV RNA concentration to be the same in serum, bile, and cultures of gallbladder epithelial cells, while HCV should be isolated from gallbladder epithelial cells, supporting this statement. Other investigators have also detected HCV RNA and HCV antigens in gallbladder specimens obtained from HCV-infected patients at the time of autopsy [41].

It is possible that viral infection of the gallbladder may increase the risk of GS formation by causing altered gallbladder mucosal function or gallbladder dysmotility; further investigations to address this interesting hypothesis are needed.

### 3.2. Chronic Alcoholism

There appears to be no definitive epidemiological evidence that alcohol affects GS formation.

Froutan et al. [42] observed that GSD disease was not significantly related to alcohol consumption. In Friedman et al.'s [43] study, there was no association between chronic alcoholism and lithogenesis. In a previous study observing the association between GS type and history of alcoholism, Trotman and Soloway [31] observed that the type of GS in patients undergoing cholecystectomy was not influenced by alcohol consumption. Bouchier [12] concluded that there were no good reasons why alcoholics should be more prone to develop gallstones.

Several other studies have confirmed that alcohol consumption reduces the risk of GS [24, 44]. Grodstein et al. [45] found a protective effect of alcohol consumption in women without cirrhosis, suggesting that a decreased risk of symptomatic GS was associated with increased alcohol intake.

On the other hand, in a study looking at the etiology of CLD, in 356 cirrhotic patients with different etiologies, being alcoholic seemed to be an important risk factor for GS formation [46]. In another study of the prevalence of GS in cirrhotic patients in relation to the etiology of disease, 180 alcoholic cirrhotic patients were compared with 320 cases of cirrhosis from other causes, and the frequency of gallstones was found to be highest in the alcoholics (33.3%) [16]. Moreover, Fornari et al. [47] followed 165 liver cirrhosis patients for 33 months and found that 28.9% with alcoholic cirrhosis developed GS during this period, but only 1.9% of viral hepatitis-cirrhosis patients developed GS.

There have been a number of studies on the association between GS formation and alcoholism in an attempt to understand the mechanism of lithogenesis [29, 48–56]. Many factors have been proposed to explain such an association, such as the direct effect of alcohol on erythrocytes [51, 52] and the biliary microenvironment [54–56], which both can cause an elevation of unconjugated bilirubin. Additionally, the enterohepatic circulation of alcohol might be related to lithogenesis [48, 49].

### 3.3. Nonalcoholic Fatty Liver Disease (NAFLD)

NAFLD is an increasingly common metabolic liver disorder that is a frequent precursor of cirrhosis and hepatocellular carcinoma [57, 58]. In previous studies, the prevalence of cholesterol GSD was shown to be higher in patients with NAFLD than in healthy subjects because they share some of the same risk factors [59–61]. The association between GS and a fatty liver was also found in a few recent papers [62–65]. Moreover, this association is more strongly apparent in females than in males [60, 63]. In addition, Fracanzani et al. [60] demonstrated that the prevalence of GSD progressively increased with advancing fibrosis and with the severity of necroinflammatory activity from GS prevalence of 15% in fibrotic stages 0–2 to 29% in stage 3 and 56% in stage 4 (cirrhosis).

Of note, the existence of an association between NAFLD and GS might stem from the shared risk factors, including obesity, type 2 diabetes mellitus, dyslipidemia, and insulin resistance [8, 66, 67]. Beyond that, in a large series of 482 Slovakian patients with metabolic risk factors, Koller et al. [68] demonstrated that NAFLD was an independent risk predictor for GSD. But Yilmaz et al. [69] demonstrated that the presence of GSD is not independently associated with definite NASH or advanced fibrosis in adult patients with biopsy-proven NAFLD. Additional cohorts or longitudinal studies are needed to identify whether the presence of NAFLD is a risk factor for GS formation and whether the prognosis will change if the patients have GSD combined with NAFLD.

### 3.4. Severity of Liver Disease

The severity of liver disease could play a role in increasing the incidence and prevalence of GSD. Several studies found that the prevalence of GSD significantly increased with Child-Pugh Class score in patients with CLD [10, 17, 20, 21, 23, 47, 70] (Table 2). This finding was confirmed in patients with HCV and patients with NAFLD [38, 60]. Considering patients with severe liver cirrhosis, the cumulative incidence of GSD among those with portosystemic shunts was found to be significantly higher than in patients who were not shunted [71].

Some data available did not find a significant difference in the prevalence of GSD according to liver function as defined by Child-Pugh score [16, 72]. In another study of the prevalence of acute or subacute liver failure and GSD in liver cirrhosis, patients with acute or subacute liver necrosis did not have increased GS formation [12]; this result is likely because the patients did not have sufficient time to develop this complication.

## 4. Pathogenesis of GS in CLD

In the human gallbladder, three types of GS exist, depending on the major constituents: pure cholesterol, pure pigment, and mixed type (small amounts of calcium and bilirubin salts) [5]. In most patients with CLD, the prevalent type is a pigment stone [29, 70, 73].

A complex pathogenesis induces the formation of GS in patients with CLD, including changes in the composition of hepatic bile, enhanced nucleation of crystals, and gallbladder hypomotility [8, 74].

### 4.1. Bile Composition

Changes in the composition of hepatic bile include reduced bile acid synthesis and transport [75], diminished cholesterol secretion, increased hydrolysis of conjugated bilirubin in the bile, and chronic hemolysis due to hypersplenism, which induces increased secretion of unconjugated bilirubin [8, 20, 70]; all of these may induce pigment lithogenesis in CLD.

### 4.2. Nucleating Factors

Apolipoprotein (apo) AI and apolipoprotein AII act as antinucleating factors in the body, and their secretion decreases when patients have CLD, possibly inducing enhanced crystal nucleation in the bile [21, 76]. In a recent study, the hepatitis B virus (HBV) X protein was shown to interact with apo AI and induce the formation of apoA-1 aggregation particles with impaired lipids, which could lead to hepatocellular cholesterol accumulation and promotion of cholesterol lithogenesis [77].

### 4.3. Gallbladder Motility

Except for the above-mentioned mechanisms of increased GS development in patients with CLD, gallbladder wall thickening and gallbladder hypomotility also play crucial roles in the formation of GS [6, 24, 75, 78]. Gallbladder wall thickening and impaired contractility have been observed in patients with liver cirrhosis, hepatic failure, and portal hypertension [78–81], providing a potential pathophysiologic basis for the high frequency of pigment stones [82]. A decrease in gallbladder motility was present in both patients with hepatitis C virus- (HCV-) related cirrhosis and those with chronic HCV hepatitis [83]. Moreover, in a retrospective cohort study of a group of 23 patients with Child-Pugh Class A score and 20 health controls, gallbladder wall thickness was increased, whereas its contractility was reduced in patients with compensated liver cirrhosis without GS [84]. Although the number of patients in the study was small, this result suggests that gallbladder hypomotility exists in patients with CLD as well as in patients with decompensated liver cirrhosis.

### 4.4. Gallbladder Absorption

Increased plasma concentrations of intestinal peptide hormones [85, 86], which inhibit gallbladder smooth muscle, and a higher resistance of the gallbladder at the receptor site might account for diminished gallbladder motility in CLD, despite the higher levels of circulating cholecystokinin (CCK) in cirrhotic patients [21, 87, 88].

## 5. Clinical Manifestations and Diagnosis

Nearly 80% of GS are asymptomatic and are discovered by ultrasonography of the right upper quadrant [4]. The typical symptom of symptomatic GS is intermittent, severe pain which starts abruptly and tends to be relieved gradually after 1–5 hours [5]. Other symptoms include abdominal discomfort and jaundice, which are similar in patients with CLD accompanied by ascites or poor liver function. Therefore, diagnosis is difficult considering symptoms only.

Signs on the main upper abdomen include tenderness and back pain. When patients have a liver tumor under the liver capsule or an enlarged liver due to alcoholic consumption, they also can have similar symptoms.

In addition, leucopenia is likely to increase because patients with CLD usually present with hypersplenism.

As can be seen from the above text, both clinical manifestation and laboratory examination of GSD might be atypical in patients with CLD. However, it is important to alert the occurrence of GS and cholecystitis if patients' symptoms cannot be explained (such as fever and abdominal pain but not associated with spontaneous peritonitis), which need further inspecting.

Because ultrasonography is a safe, noninvasive, low-cost procedure, with greater than 95% specificity and sensitivity, it has become the best method for diagnosing GSD [89]. Ultrasonographic features of cholelithiasis were as follows: the presence of movable echogenic structure(s) within the gallbladder lumen which cause a posterior acoustic shadow. However, due to the location of GS and the interruption of intestinal gas, ultrasonography has limited value in detecting small bile duct stones and in the diagnosis of choledocholithiasis.

In summary, we can see that the diagnosis of GSD can be easily confused with clinical symptoms of liver disease in patients with CLD. Clinicians should be aware of such a diagnostic challenge.

## 6. Treatment

Treatment of asymptomatic cholelithiasis in CLD patients does not generally include prophylactic cholecystectomy, because the risk of stones causing symptoms or complications is low and the risk of surgery is higher than in other patients without CLD [8].

In a study of 64 patients with liver cirrhosis, 33 cirrhotic patients with asymptomatic cholelithiasis underwent cholecystectomy or cholecystolithotomy during portal diversion; the mortality and morbidity rates in these patients were not significantly different within another cohort of 170 cirrhotic patients who underwent portal operation alone [90]. However, in another study, all cirrhotic patients with GS required blood transfusion during elective surgical treatment [91]. Currently, more randomized studies must be done to evaluate better approaches for CLD patients with asymptomatic GS, such as observation alone or elective cholecystectomy.

The main treatments for symptomatic GS in CLD are laparoscopic cholecystectomy (LC) and open cholecystectomy (OC), the aims of which are to relieve symptoms and prevent serious complications [4].

Patients with cirrhosis are more likely to undergo cholecystectomy for emergent reasons compared to those who do not have liver disease [92]. The postoperative mortality in cirrhotic patients who undergo OC is 7.5%–25.5% [90, 93–95]. Wound infections, bile leaks, pulmonary embolisms, liver bleeding, and cardiopulmonary issues are the main complications [92]. The first study evaluating the outcome of LC in cirrhotic patients was published in 1993 [96]. Compared with OC, LC has less operative blood loss, shorter operative and recovery times, reduced hospital stays, and reduced complications rates in CLD patients [92, 97–99].

The best predictors of outcome after LC would be the Child-Pugh and MELD scores [100, 101].

The morbidity and mortality rates after LC have been shown to be acceptable in Child-Pugh Classes A and B patients with symptomatic GS [102–107]. However, the risk of mortality and complications for cholecystectomy in patients with Child-Pugh Class C are high [108]. These patients are more prone to complications such as acute liver failure, encephalopathy, adult respiratory distress syndrome, acute renal failure, and sepsis than other patients with Child-Pugh Class A or B [101]. Because of this, no conclusions can be drawn regarding the outcome of LC due to the lack of data.

## Figures and Tables

**Table 1:** Gallstone prevalence in chronic liver disease.

First author	Year	Type of study	Number of subjects	Prevalence of gallstones (%)
Bouchier	1969	Autopsy	Patients with CLD (*n* = 235)	29.4
			Controls (*n* = 4460)	12.8
Goebell	1981	Autopsy	Patients with CLD (*n* = 697)	21.5
			Controls (*n* = 11143)	16.5
Segala	1991	US	Patients with CLD	29.4
Fornari	1994	US	Patients with CLD (*n* = 410)	31.9
			Controls (*n* = 414)	20.7
Acalovschi	2009	US	Patients with CLD (*n* = 453)	19
			Controls (*n* = 879)	17

**Table 2:** Prevalence of gallstones (%) in cirrhotic patients according to Child-Pugh score.

First author	Year	Number of subjects	Class A	Class B	Class C
Elzouki	1999	67	19	26	56
Conte	1999	1010	24	28	37
Fornari	1990	410	15.7	37.8	37.6
Fornari	1994	165	6.4	24	48
